# Supplementary material for: Early-life telomere length variation under changing developmental conditions in long-lived bats
Source: Philos Trans R Soc Lond B Biol Sci. 2026 Nov 12;381(1963):20250338. doi: 10.1098/rstb.2025.0338 (PMC13755923; doi:10.1098/rstb.2025.0338)
Supplement: Supplementary file 1 [file rstb.2025.0338.zip › rstb.2025.0338/rstb20250338_si_001.pdf]

Early-life telomere length variation under changing developmental conditions in long-lived bats.

Tadhg Lonergan[1†], Megan L. Power[1,2†], Luke Romaine[2], Roger Ransome[2], Frederic Touzalin[1,3], Sébastien J. Puechmaille[4,5], Gareth Jones[2], Emma C. Teeling[1*]

[1]School of Biology and Environmental Science, University College Dublin, Dublin, Ireland; [2] School of Biological Sciences, University of Bristol, Bristol, UK; [3]Department of Biosciences, University of Exeter, Exeter, UK; [4] Department of Biosciences, University of Exeter, Exeter, UK; [5] Institut des Sciences de l'Evolution de Montpellier, Universite de Montpellier, CNRS, IRD, Montpellier, France; [6]Institut Universitaire de France, Paris, France.

*Author for correspondence (emma.teeling@ucd.ie)

†T.L. and M.L.P. are joint first authors

‡ G.J. and E.C.T. are joint senior authors.

Supplementary methods

To amplify telomere sequences, primers tel1 (5′-GGTTTTTGAGGGTGAGGGTGAGGGTGAGGGTGAGGGT-3′) and tel2 (5′-TCCCGACTATCCCTATCCCTATCCCTATCCCTATCC-3′) were used (1). The single copy gene, BDNF (mammalian brain-derived neurotrophic factor), was amplified using primers BDNF_F1 (5′-AGCTGAGCGTATGTGACAGT-3′) and BDNF_R1 (5′-TGGGATTACACTTGGTCTCGT-3′) from (2). To minimise potential confounding between qPCR plate effects and biological variables such as sampling year, *Myotis myotis* samples were organised using a slicing approach (3). This method ensures samples from different years were deliberately distributed across plates rather than grouped sequentially or randomly. By "slicing" across sampling years, this allows for the statistical separation of technical variation (e.g., plate effects) from biological variation such as cohort or environmental effects.

For *Rhinolophus ferrumequinum*, samples were randomly allocated onto extractions plates along with other samples collected as part of this long-term study (4,5). Although randomisation minimises confounding between biological variables (e.g. sampling year) and batch effects, residual between-plate variation can occur in qPCR assays (3). Therefore, qPCR plate identity was included as a covariate in all statistical models to account for technical variance.

For both species, reactions were carried out in 10µl volumes with 5µl of Takara SYBR Green (Clontech), 0.2µl of ROX, 2.5µl of DNA with the remaining volume made up with PCR grade water (Invitrogen). Telomere and SCG reactions were the same except for primer volumes with all primers used at 50µM concentration (0.027µl tel1, 0.09µl tel2, 0.12µl BDNF_F1, 0.2µl BDNF_R1). For baseline correction, calculating amplification efficiencies and Cq values (the number of cycles the qPCR amplification curve crosses a set fluorescence threshold), the software package LinRegPCR was used (6). After normalization using the golden sample as reference, the average Cq was calculated across the replicates for each sample for both amplicons. rTL was calculated for each sample, following (7):

$$rTL = \frac{(E_{TEL})^{Cq_{TEL(Calibrator)} - Cq_{TEL(Sample)}}}{(E_{SCG})^{Cq_{SCG(Calibrator)} - Cq_{SCG(Sample)}}}$$

Where E_{TEL} and E_{SCG} are the amplicon and plate specific qPCR efficiencies, $Cq_{TEL(Calibrator)}$ and $Cq_{SCG(Calibrator)}$ are the average Cq values for the "golden sample" calibrator for each amplicon and $Cq_{TEL(Sample)}$ and $Cq_{SCG(Sample)}$ are the average Cq values for individual samples for each amplicon respectively.

Scaled Mass Index

For both species, models included scaled mass index (SMI) as a fixed effect. SMI was calculated following (8) as:

$$SMI = W_i \left(\frac{L_0}{L_i}\right) b$$

where W_i is body mass, L_i is forearm length (as a measure of structural size), L_0 is the mean forearm length of the sampled population, and b is the scaling exponent estimated from an ordinary least squares regression of log-transformed mass on log-transformed forearm length. Forearm length was used as the linear body size metric.

Table S1: Summary of the *Rhinolophus ferrumequinum* samples caught during early-life between 2016 and 2020 in the Woodchester Mansion population. There were 847 rTL measurements taken from 441 individuals.

Rhinolophus ferrumequinum			
	**Number of samples**	**Number of individuals**	
	**1**	72	
	**2**	306	
	**3**	63	
**Number of samples by year**		**Number of samples by month**	
**2016**	149	**June**	100
**2017**	233	**July**	333
**2018**	164	**August**	293
**2019**	172	**September**	147
**2020**	155		

Table S2: Summary of mixed-effects model testing the effect of sample storage time (days) and DNA storage time (days) on *Myotis myotis* rTL. Significant values (p < 0.05) are in bold.

Effects	Estimate	Std Error	t value	p-value
**Fixed Effects**				
(Intercept)	0.072	0.265	0.270	0.794
Sample storage time	0.010	0.165	0.058	0.954
DNA storage time	-0.063	0.079	-0.803	0.423
**Random Effects**	**Variance**			
Year	0.257			
qPCR Plate	0.234			
Residual	0.599			

Table S3: Summary of mixed-effects model testing the effect sample storage time (days) and DNA storage time (days) on *Rhinolophus ferrumequinum* rTL. Significant values (p < 0.05) are in bold.

Effects	Estimate	Std Error	t value	p-value
**Fixed Effects**				
(Intercept)	-0.061	0.165	-0.367	0.781
Age (in days)	**-0.187**	**0.033**	**-5.731**	**<0.001**
Age (in days)^2	**0.103**	**0.034**	**3.039**	**0.003**
Sample storage time	0.089	0.087	1.022	0.311
DNA storage time	-0.045	0.047	-0.954	0.341
**Random Effects**	**Variance**			
Year	0.106			
qPCR Plate	0.261			
Individual identity	0.097			
Residual	0.479			

Table S4: Summary of mixed-effects model testing the effect of birth year cohort (factor), sex, and scaled mass index (SMI) on juvenile telomere length (z-scores) in *Myotis myotis*. Reference year is 2014. Significant values ($p < 0.05$) are in bold.

Effects	Estimate	Std Error	t value	p-value
**Fixed Effects**				
(Intercept, Year 2014)	-0.239	0.515	-0.464	0.644
Year 2015	-0.012	0.154	-0.078	0.938
Year 2016	0.018	0.156	0.118	0.907
Year 2017	0.029	0.159	0.182	0.855
Year 2018	0.196	0.155	1.260	0.208
Year 2019	0.098	0.162	0.608	0.544
**Year 2020**	**1.422**	**0.164**	**8.699**	**<0.001**
Year 2021	0.281	0.166	1.695	0.091
**Year 2022**	**-0.761**	**0.179**	**-4.240**	**<0.001**
Year 2023	-0.077	0.183	-0.417	0.677
Sex (Male)	0.084	0.073	1.157	0.248
SMI	0.007	0.022	0.304	0.761
**Random Effects**	**Variance**			
qPCR Plate	0.238			
Residual	0.586			

Table S5: Summary of mixed-effects model testing the effect of birth year cohort (factor), sex, age in days (quadratic function) and scaled mass index (SMI) on juvenile telomere length in *Rhinolophus ferrumequinum* (z-scores). Reference year is 2016. Significant values ($p < 0.05$) are in bold.

Effects	Estimate	Std Error	t value	p-value
**Fixed Effects**				
(Intercept, Year 2016)	-0.463	0.457	-1.013	0.311
Year 2017	0.341	0.210	1.619	0.110
Year 2018	0.032	0.223	0.144	0.886
Year 2019	-0.149	0.231	-0.644	0.522
**Year 2020**	**-0.946**	**0.250**	**-3.789**	**<0.001**
Sex (Male)	0.028	0.059	0.474	0.636
SMI	0.042	0.034	1.233	0.218
**Age (in days)**	**-5.375**	**0.940**	**-5.721**	**<0.001**
**Age (in days)^2**	**2.880**	**0.978**	**2.945**	**0.003**
**Random Effects**	**Variance**			
qPCR Plate	0.236			
Individual identity	0.093			
Residual	0.481			

Table S6. Summary of the mixed-effects model partitioning age into within-individual (Delta age) and between-individual (Average age) and their respective quadratic components using within-subject centring in juvenile *Rhinolophus ferrumequinum* (rTL z-scores). The model included birth year cohort (factor), sex and scaled mass index (SMI) as fixed effects, with individual identity and qPCR plate fitted as random intercepts. Reference year is 2016. Significant values ($p < 0.05$) are shown in bold.

Effects	Estimate	Std Error	t value	p-value
**Fixed Effects**				
(Intercept, Year 2016)	-0.292	0.493	-0.631	0.528
Year 2017	0.318	0.218	1.459	0.149
Year 2018	0.037	0.229	0.165	0.870
Year 2019	-0.120	0.239	-0.501	0.618
**Year 2020**	**-0.942**	**0.257**	**-3.668**	**0.001**
Sex (Male)	0.031	0.059	0.526	0.599
SMI	0.028	0.034	0.822	0.411
Average age	-0.927	1.129	-0.821	0.412
Average age ^{2}	1.191	1.040	1.146	0.252
**Delta age**	**-4.621**	**0.853**	**-5.417**	**<0.001**
**Delta age ^{2}**	**2.705**	**1.072**	**2.524**	**0.012**
**Random Effects**	**Variance**			
qPCR Plate	0.258			
Individual identity	0.094			
Residual	0.477			

Table S7: Summary of sliding window analysis for early-life rTL in relation to climatic variables (temperature and rainfall) for *Myotis myotis*. Different statistical functions (linear or quadratic) were used to model the relationships, and ΔAICc values indicate the relative model support compared to the null model. "WindowOpen" and "WindowClose" represent the days before sampling where climate variables were measured within a selected sliding window.

Myotis myotis						
**Response variable**	**Climate**	**stat**	**func**	**DeltaAICc**	**WindowOpen**	**WindowClose**
Juvenile rTL	Temp	mean	lin	-5.92	59	54
	Rain	mean	lin	-11.84	28	18
	Temp	mean	quad	-7.43	41	36
	Rain	mean	quad	-19.29	24	15

Table S8. Results of the linear mixed-effects models analysing the relationship between juvenile rTL in *Myotis myotis* and rainfall using both the highest-ranked climatic window identified by the climwin sliding window analysis and the corresponding median window derived from the 95% model confidence set. Models included sex and scaled mass index (SMI) as fixed effects, with qPCR plate fitted as a random intercept. Significant p-values ($p < 0.05$) are shown in bold.

**Best supported window (24 to 15 days)**				
**Effects**	**Estimate**	**Std Error**	**t value**	**p-value**
**Fixed effects**				
(Intercept)	-0.352	0.474	-0.743	0.460
Sex (Male)	0.080	0.073	1.092	0.276
Scaled Mass Index (SMI)	0.016	0.020	0.788	0.432
**Rainfall**	**6.471**	**1.041**	**6.219**	**<0.001**
**Rainfall^2**	**8.605**	**1.034**	**8.320**	**<0.001**
**Random effects**				
qPCR Plate	0.201			
Year	0.009			
Residual	0.598			
**Median window (32 to 16 days)**				
**Fixed effects**				
(Intercept)	-0.113	0.512	-0.221	0.825
Sex (Male)	0.087	0.073	1.192	0.234
Scaled Mass Index (SMI)	0.005	0.022	0.246	0.806
**Rainfall**	**6.974**	**2.038**	**3.423**	**0.007**
**Rainfall^2**	**6.226**	**2.043**	**3.047**	**0.013**
**Random effects**				
qPCR Plate	0.226			
Year	0.071			
Residual	0.598			

Table S9: Median climatic windows derived from the 95% model confidence set for each climate variable in *Myotis myotis* and *Rhinolophus ferrumequinum*. Median opening (WindowOpen) and closing (WindowClose) dates were calculated from all windows comprising 95% of the Akaike model weights, and corresponding ΔAICc values are shown for single-window models refitted using these median windows.

Response variable	Climate	stat	func	DeltaAICc	WindowOpen	WindowClose
Myotis myotis						
Juvenile rTL	Temp	mean	lin	1.678	44	16
	Rain	mean	lin	-1.740	37	14
	Temp	mean	quad	2.539	40	18
	Rain	mean	quad	-7.016	32	16
Rhinolophus ferrumequinum						
Juvenile rTL	Temp	mean	lin	-12.520	28	14
	Rain	mean	lin	-25.104	8	1
	Temp	mean	quad	-22.382	29	17
	Rain	mean	quad	-25.969	15.5	9

Table S10. Results of the linear mixed-effects models analysing the relationship between juvenile rTL in *Myotis myotis* and temperature using both the highest-ranked climatic window identified by the climwin sliding window analysis and the corresponding median window derived from the 95% model confidence set. Models included sex and scaled mass index (SMI) as fixed effects, with qPCR plate fitted as a random intercept. Significant p-values ($p < 0.05$) are shown in bold.

**Best supported window (41 to 36 days)**				
**Effects**	**Estimate**	**Std Error**	**t value**	**p-value**
**Fixed effects**				
(Intercept)	-0.102	0.511	-0.200	0.841
Sex (Male)	0.090	0.073	1.227	0.220
Scaled Mass Index (SMI)	0.005	0.022	0.214	0.831
**Average Temperature**	**6.812**	**1.977**	**3.446**	**0.008**
**Average Temperature^2**	**6.679**	**1.938**	**3.446**	**0.008**
**Random effects**				
qPCR Plate	0.233			
Year	0.064			
Residual	0.598			
**Median window (40 to 18 days)**				
**Fixed effects**				
(Intercept)	-0.117	0.538	-0.217	0.828
Sex (Male)	0.084	0.074	1.146	0.252
Scaled Mass Index (SMI)	0.007	0.022	0.301	0.764
Average Temperature	-4.521	3.122	-1.310	0.220
Average Temperature^2	-0.366	3.448	-0.106	0.918
**Random effects**				
qPCR Plate	0.253			
Year	0.222			
Residual	0.597			

Table S11. Sensitivity analysis of the *Rhinolophus ferrumequinum* sliding window analysis using 30-, 45- and 60-day search intervals. For each search interval, the highest-ranked climatic window is shown for temperature and rainfall. ΔAICc values indicate model support relative to the baseline model. WindowOpen and WindowClose represent the opening and closing days before sampling defining each climatic window.

			***Rhinolophus ferrumequinum* (30 days)**			
**Response variable**	**Climate**	**stat**	**func**	**DeltaAICc**	**WindowOpen**	**WindowClose**
Juvenile rTL	Temp	mean	lin	-17.75	28	21
	Rain	mean	lin	-25.10	8	1
	Temp	mean	quad	-24.23	30	15
	Rain	mean	quad	-30.60	30	25
			***Rhinolophus ferrumequinum* (45 days)**			
Juvenile rTL	Temp	mean	lin	-17.75	28	21
	Rain	mean	lin	-25.10	8	1
	Temp	mean	quad	-27.91	38	4
	Rain	mean	quad	-30.60	30	25
			***Rhinolophus ferrumequinum* (60 days)**			
Juvenile rTL	Temp	mean	lin	-21.84	55	50
	Rain	mean	lin	-25.10	8	1
	Temp	mean	quad	-27.91	38	4
	Rain	mean	quad	-30.60	30	25

Table S12. Results of the linear mixed-effects models analysing the relationship between juvenile rTL in *R. ferrumequinum* and rainfall using both the highest-ranked climatic window identified by the climwin sliding window analysis and the corresponding median window derived from the 95% model confidence set. Models included age in days (modelled as a quadratic term), sex and scaled mass index (SMI) as fixed effects, with qPCR plate, year sampled and individual identity treated as a random effect. Significant p-values (<0.05) are highlighted in bold.

**Best supported window (30 to 25 days)**				
**Effects**	**Estimate**	**Std Error**	**t value**	**p-value**
**Fixed effects**				
(Intercept)	-0.603	0.468	-1.288	0.201
**Age (in days)**	**-4.223**	**0.972**	**-4.344**	**<0.001**
Age (in days)^2	1.969	1.080	1.823	0.069
Sex (Male)	0.033	0.058	0.562	0.575
Scaled Mass Index (SMI)	0.042	0.033	1.269	0.205
Rainfall	2.976	1.864	1.596	0.112
**Rainfall^2**	**8.313**	**1.590**	**5.230**	**<0.001**
**Random effects**				
qPCR Plate	0.247			
Individual identity	0.100			
Year	0.195			
Residual	0.453			
**Median window (15.5 to 9 days)**				
**Fixed effects**				
(Intercept)	-0.710	0.459	-1.548	0.124
**Age (in days)**	**-4.848**	**1.097**	**-4.419**	**<0.001**
**Age (in days)^2**	**3.556**	**1.181**	**3.010**	**0.003**
Sex (Male)	0.028	0.058	0.480	0.631
Scaled Mass Index (SMI)	0.052	0.033	1.555	0.120
**Rainfall**	**-2.371**	**1.196**	**-1.982**	**0.048**
**Rainfall^2**	**4.620**	**1.103**	**4.189**	**<0.001**
**Random effects**				
qPCR Plate	0.255			
Individual identity	0.098			
Year	0.139			
Residual	0.458			

Table S13: Results of the linear mixed-effects models analysing the relationship between juvenile rTL in *R. ferrumequinum* and temperature during the window identified by the climwin sliding window analysis. Model includes age in days (modelled as a quadratic term), sex and scaled mass index (SMI) as fixed effects, with qPCR plate, year sampled and individual identity treated as a random effect. Significant p-values (<0.05) are highlighted in bold.

Effects	Estimate	Std Error	t value	p-value
**Fixed effects**				
(Intercept)	-0.745	0.477	-1.562	0.122
**Age (in days)**	**-5.640**	**0.926**	**-6.093**	**<0.001**
**Age (in days)^2**	**3.499**	**0.989**	**3.538**	**<0.001**
Sex (Male)	0.029	0.058	0.497	0.619
Scaled Mass Index (SMI)	0.055	0.034	1.633	0.103
Average Temperature	-1.102	1.828	-0.603	0.547
**Average Temperature^2**	**-6.094**	**1.480**	**-4.117**	**<0.001**
**Random Effects**	**Variance**			
qPCR Plate	0.250			
Individual identity	0.097			
Year	0.229			
Residual	0.598			

Table S14: Results of the linear mixed-effects model investigating the relationship between early-life rTL of *Myotis myotis* juveniles and maternal age at conception (MAC). Significant values ($p<0.05$) are highlighted in bold.

Effects	Estimate	Std Error	t value	p-value
**Fixed Effects**				
(Intercept)	-0.262	0.573	-0.457	0.648
MAC	0.051	0.027	1.872	0.062
Sex (Male)	0.173	0.156	1.106	0.270
Scaled Mass Index	0.011	0.025	0.454	0.650
MAC * Sex (Male)	-0.042	0.038	-1.107	0.269
**Random Effects**	**Variance**			
Mother identity	0.056			
Year	0.181			
qPCR Plate	0.214			
Residual	0.443			

Table S15: Summary of mixed-effects model testing between and within mother at conception effects in *Myotis myotis*. Significant values ($p<0.05$) are highlighted in bold.

Effects	Estimate	Std Error	t value	p-value
**Fixed Effects**				
(Intercept)	-0.299	0.581	-0.514	0.608
Mean MAC	0.055	0.031	1.775	0.077
Delta MAC	0.038	0.054	0.699	0.485
Sex (Male)	0.156	0.174	0.898	0.370
SMI	0.012	0.025	0.501	0.617
Sex (Male)*Mean MAC	-0.038	0.044	-0.858	0.392
Sex (Male)*Delta MAC	-0.054	0.076	-0.714	0.476
**Random Effects**	**Variance**			
qPCR Plate	0.215			
Mother identity	0.054			
Year	0.187			
Residual	0.444			

Table S16: Results of the linear mixed-effects model investigating the relationship between early-life rTL of *Rhinolophus ferrumequinum* juveniles and maternal age at conception (MAC). Significant values ($p<0.05$) are highlighted in bold.

Effects	Estimate	Std Error	t value	p-value
**Fixed Effects**				
(Intercept)	-0.597	0.479	-1.246	0.215
**Age (in days)**	**-5.414**	**0.937**	**-5.778**	**<0.001**
**Age (in days)²**	**2.687**	**0.981**	**2.739**	**0.006**
MAC	0.002	0.010	0.210	0.834
Sex (Male)	0.055	0.115	0.480	0.632
SMI	0.040	0.035	1.142	0.254
Sex (Male)*MAC	-0.004	0.014	-0.297	0.766
**Random Effects**	**Variance**			
qPCR Plate	0.265			
Individual identity	0.068			
Mother identity	0.033			
Year	0.156			

Table S17: Summary of mixed-effects model testing between and within mother age at conception (MAC) effects in *Rhinolophus ferrumequinum*. Significant values ($p<0.05$) are highlighted in bold.

Effects	Estimate	Std Error	t value	p-value
**Fixed Effects**				
(Intercept)	-0.517	0.486	-1.062	0.290
**Age (in days)**	**-5.414**	**0.936**	**-5.783**	**<0.001**
**Age (in days)²**	**2.649**	**0.980**	**2.703**	**0.007**
Mean MAC	-0.004	0.011	-0.393	0.695
Delta MAC	0.099	0.055	1.809	0.071
Sex (Male)	0.008	0.116	0.068	0.946
SMI	0.037	0.035	1.045	0.296
Sex (Male)*Mean MAC	0.003	0.014	0.228	0.820
**Sex (Male)*Delta MAC**	**-0.121**	**0.053**	**-2.275**	**0.024**
**Random Effects**	**Variance**			
qPCR Plate	0.265			
Individual identity	0.059			
Mother identity	0.037			
Year	0.188			
Residual	0.478			

Table S18: Fixed, random and variance-covariance matrices from a bivariate model of rTL and first year survival in female *Myotis myotis*. Covariance and correlations were estimated at the yearly and among-individual levels. Estimates are given as the mode of the posterior distribution with 95% Highest Posterior Density credible intervals. For variance-covariance matrices, variances are shown on the diagonal, covariances in the bottom left and correlations in the top right. Significant covariances and correlations are shown in bold.

Response variables: rTL, Survival	Posterior mode	95% CI	Effective sample size	pMCMC
Fixed effects (n = 496)				
rTL				
Intercept	-0.205	[-2.048, 1.256]	2651	0.705
SMI	0.021	[-0.048, 0.083]	3000	0.563
Survival				
Intercept	-1.929	[-3.796, 1.145]	3000	0.245
SMI	0.005	[-0.043, 0.183]	3000	0.299
Random effects				
qPCR plate	0.280	[0.110, 2.198]		
Variance-covariance matrices				
**Year sampled**	*rTL*	*Survival*		
rTL	0.314 [0.169, 1.260]	0.461 [-0.188, 0.825]		
Survival	0.139 [-0.234, 0.941]	0.486 [0.197, 2.104]		
**Residual**	*rTL*	*Survival*		
rTL	0.577 [0.490, 0.700]	-0.017 [-0.188, 0.170]		
Survival	-0.013 [-0.153, 0.124]	1.000 [1.000, 1.000]		

Table S19: Results of a bivariate Bayesian mixed-effects model jointly analysing juvenile relative telomere length (rTL) and first-year survival in female *Rhinolophus ferrumequinum*. Estimates are given as the mode of the posterior distribution with 95% Highest Posterior Density credible intervals. rTL was modelled as a Gaussian trait with repeated measures, while survival to the following summer was modelled as a threshold (binary) trait measured once per individual. Random effects for qPCR plate and year were fitted for rTL only (*) to account for technical and temporal variation. Individual identity was specified with an unstructured (co)variance matrix, allowing estimation of among-individual variance in rTL, variance in individual rTL slopes, variance in survival, and their covariances. Correlation coefficients (r) are also shown. Significant covariances and correlations are shown in bold.

**Response variables: rTL, Survival**	**Posterior mode**	**95% CI**	**Effective sample size**	**pMCMC**
Fixed effects (n = samples, N = individuals)				
rTL				
Intercept	-1.493	[-3.027, 0.320]	3000	0.116
Age (centered)	-0.012	[-0.027, 0.008]	3000	0.323
SMI	0.105	[-0.021, 0.222]	3000	0.092
Survival				
Intercept	-0.208	[-1.372, 0.695]	3109	0.531
SMI	0.051	[-0.022, 0.141]	3084	0.175
Random effects				
qPCR plate*	0.169	[0.085, 0.349]	3000	
Year*	0.297	[0.092, 1.648]	3200	
Residual	0.187	[0.123, 0.294]	2756	
**Individual identity**				
V_{rTL}	0.292	[0.202, 0.402]		
$V_{rTL:Slope}$	0.015	[0.013, 0.018]		
V_{Surv}	0.253	[0.210, 0.308]		
$COV_{rTL,rTL:Slope}$	-0.001	[-0.010, 0.009]		
$COV_{rTL,Surv}$	-0.006	[-0.055, 0.046]		
$COV_{rTL:Slope,Surv}$	-0.001	[-0.010, 0.008]		
$r_{(rTL,rTL:Slope)}$	-0.014	[-0.148, 0.126]		
$r_{(rTL,Surv)}$	0.011	[-0.190, 0.167]		
$r_{(rTL:Slope,Surv)}$	-0.013	[-0.149, 0.123]		

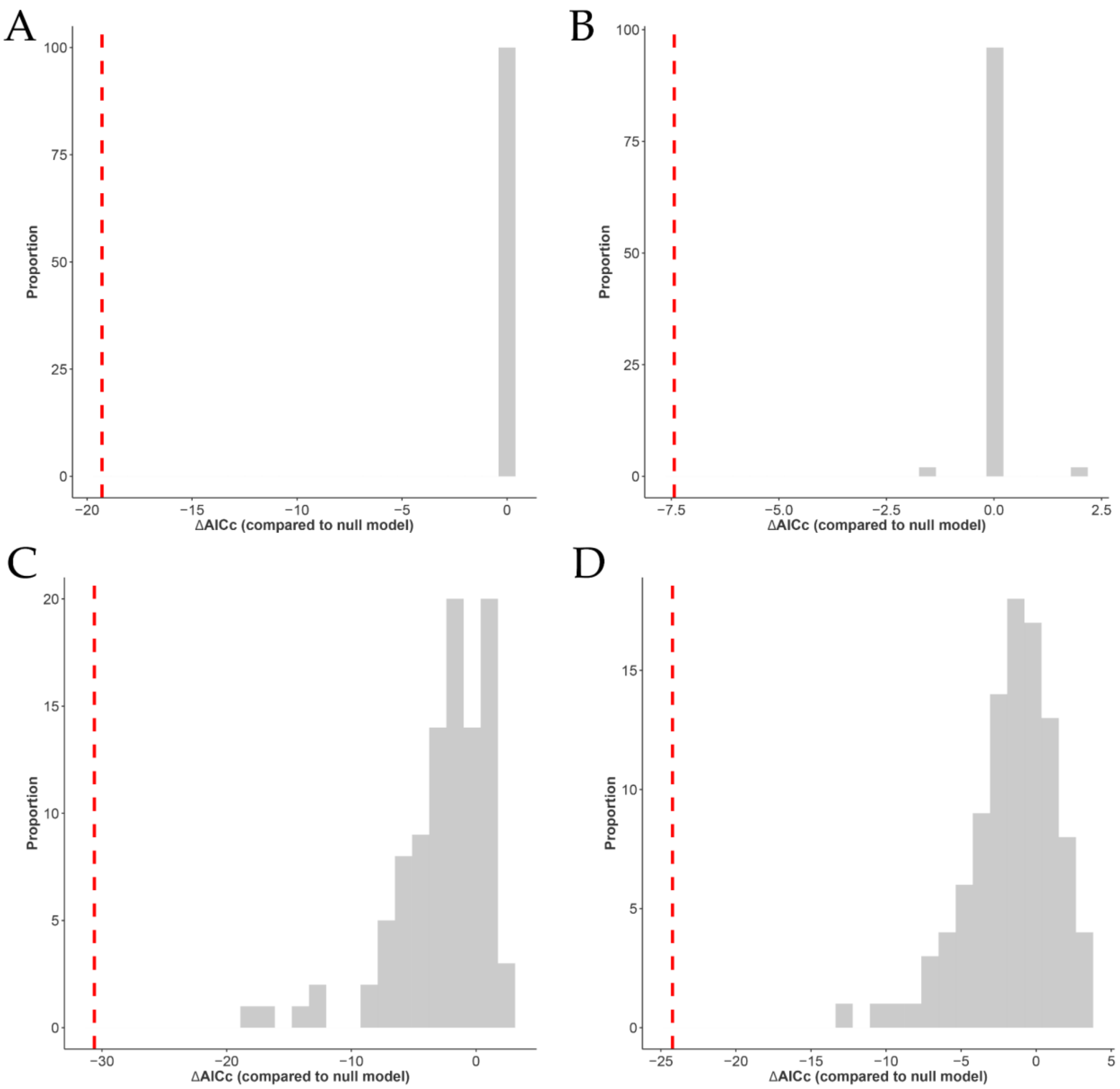

Figure S1: Histograms representing the best AIC support for 100 randomised sliding window analyses for *Myotis myotis* (A) average daily rainfall (quadratic) and (B) average daily temperature (quadratic) analyses and *Rhinolophus ferrumequinum* (C) average daily rainfall (quadratic) and (D) average daily temperature (quadratic) analyses. Red dashed lines represent the AIC support achieved with the real data.

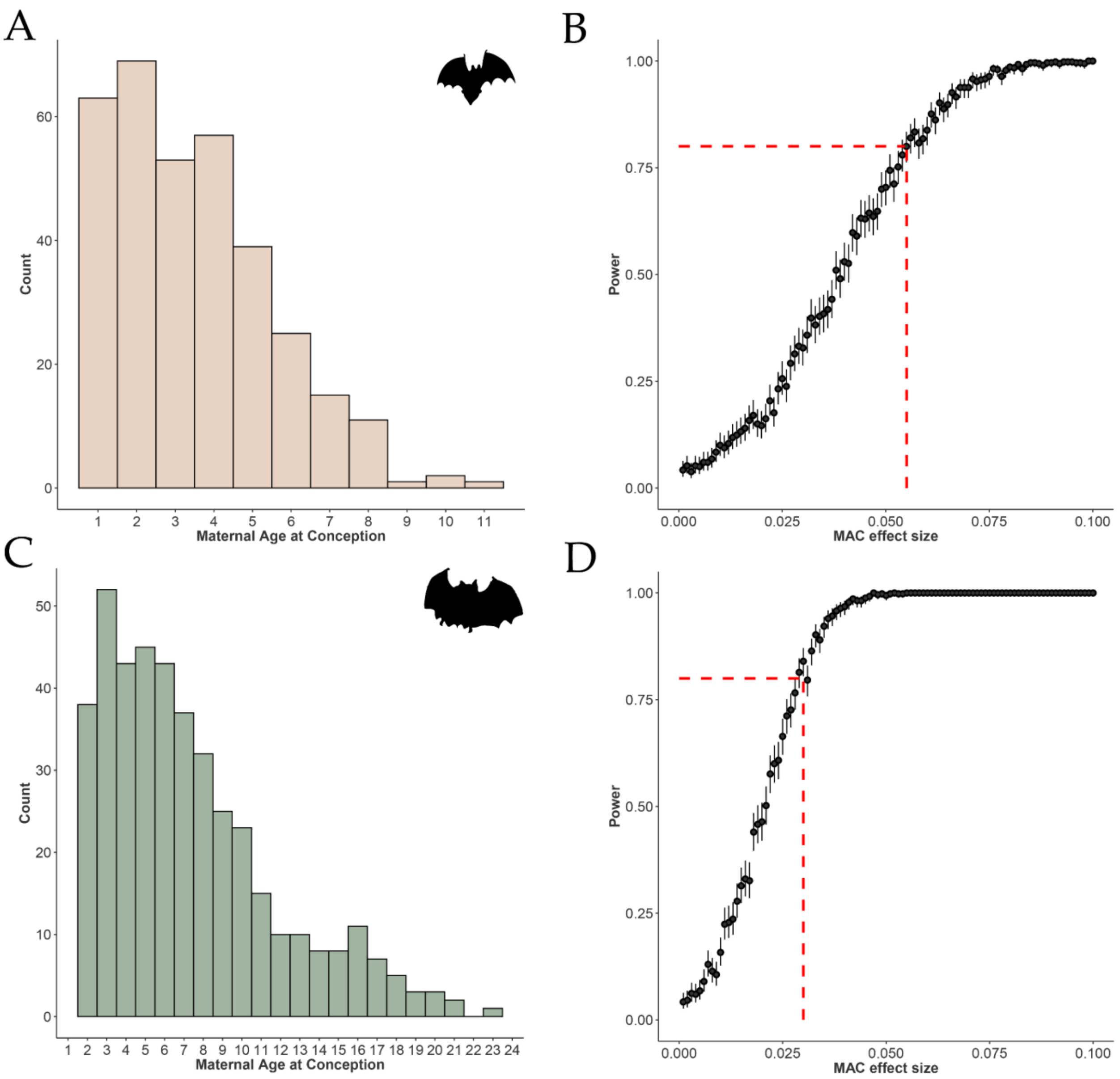

Figure S2: Distribution of maternal ages and power analysis for detecting maternal age effects on juvenile *Myotis myotis* and *Rhinolophus ferrumequinum* telomere length. (A) Histogram showing the distribution of maternal age at conception (MAC) for *M. myotis*. Maternal identity was assigned to 336 juveniles in this study, with maternal age estimated at the time of offspring conception. (B) Simulation-based power analysis for detecting MAC effects on juvenile relative telomere length in *M. myotis*. Power curves show the probability of detecting effects of increasing magnitude given the observed data and model structure. Red dashed lines indicate the effect size detectable with ≥80% power (MAC ≥ 0.05), corresponding to a correlation coefficient of r = 0.086. (C) Distribution of maternal age at conception for *R. ferrumequinum*. Maternal identity was assigned to 422 juveniles in this study. (D) Simulation-based power analysis for *R. ferrumequinum*, showing ≥80% power to detect MAC effect sizes ≥ 0.030, equivalent to a correlation coefficient of r = 0.131. Error bars represent 95% confidence intervals around simulated power estimates.

Supplementary references

1. Cawthon, R. M. (2002). Telomere measurement by quantitative PCR. *Nucleic Acids Res.*, 30(10), e47-e47.
2. Foley, N. M. et al., (2018). Growing old, yet staying young: the role of telomeres in bats' exceptional longevity. *Sci. Adv.*, 4(2), eaao0926.
3. Van Lieshout, S.H., Froy, H., Schroeder, J., Burke, T., Simons, M.J., Dugdale, H.L. (2020). Slicing: A sustainable approach to structuring samples for analysis in long-term studies. *Methods Ecol. Evol.*, 11(3), 418-430.
4. Power, M. L., Ransome, R. D., Riquier, S., Romaine, L., Jones, G., & Teeling, E. C. (2023). Hibernation telomere dynamics in a shifting climate: insights from wild greater horseshoe bats. *Proc. R. Soc. Lond. B Biol. Sci.*, 290(2008), 20231589.
5. Power, M. L., Ransome, R. D., Romaine, L., Jones, G., & Teeling, E. C. (2025). Short-and long-term costs of reproduction revealed by telomere dynamics in wild greater horseshoe bats. *PNAS*, 122(50), e2515125122.
6. Ruijter, J. M., Ramakers, C., Hoogaars, W. M. H., Karlen, Y., Bakker, O., Van den Hoff, M. J. B., & Moorman, A. (2009). Amplification efficiency: linking baseline and bias in the analysis of quantitative PCR data. *Nucleic Acids Res.*, 37(6), e45-e45.
7. Pfaffl, M. W. (2001). A new mathematical model for relative quantification in real-time RT–PCR. *Nucleic Acids Res.*, 29(9), e45-e45.
8. Peig, J., Green, A.J. (2009). New perspectives for estimating body condition from mass/length data: the scaled mass index as an alternative method. *Oikos*, 118(12), 1883-1891.
